# Enterovirus 75 and Aseptic Meningitis, Spain, 2005

**DOI:** 10.3201/eid1210.060353

**Published:** 2006-10

**Authors:** Ana Avellón, Gurutze Rubio, Gustavo Palacios, Inmaculada Casas, Nuria Rabella, Gabriel Reina, Cármen Pérez, W. Ian Lipkin, Gloria Trallero

**Affiliations:** * Carlos III Institute of Health, Majadahonda, Spain;; † Cruzes Hospital, Bilbao, Spain;; ‡ Columbia University, New York, New York, USA;; § Sant Pau y Santa Creu Hospital, Barcelona, Spain;; ¶ Virgen de las Nieves Hospital, Granada, Spain;; # Doctor Negrín Hospital, Las Palmas de Gran Canaria, Spain

**Keywords:** Enterovirus, Enterovirus type 75, EV75, Aseptic meningitis, letter

**To the Editor:** Although most human enterovirus (EV) (genus *Enterovirus*, family *Picornaviridae*) infections are asymptomatic, they can cause upper respiratory illness, febrile rash, aseptic meningitis, pleurodynia, encephalitis, acute flaccid paralysis, and neonatal sepsislike disease (*1*). Most EVs have been implicated in aseptic meningitis, most notably echovirus (E) 30, 9, 6, and 11 and coxsackie B virus (CBV) type 5 (*2*); other serotypes are less frequently associated with neurologic disease.

New EV serotypes have come to light, chiefly as a result of molecular typing methods (*3**–**6*). EV75 was proposed as a new serotype of the EV genus in 2004 (*5*). Retrospective analysis showed that it had circulated sporadically in Asia, the United States, and Africa since at least 1974. Only 8 isolates of this serotype have been reported worldwide, in 1974, 1985, 1986, 1987 (n = 2), 1998, and 2000 (n = 2). Infection in those cases was associated with respiratory disease, acute flaccid paralysis, neonatal jaundice, failure to thrive, or unspecified neurologic disease or was asymptomatic. At the time of writing this manuscript, EV75 had not been linked to aseptic meningitis.

From May 2005 through January 2006, 106 EVs were received for typing from Spanish hospital laboratories; 46 of them were from patients with aseptic meningitis, 10 from patients or contacts of patients with acute flaccid paralysis, 27 from patients with fever, 7 from patients with respiratory diseases, and 16 from other patients. Twenty EVs could not be typed by serum neutralization (*7*); however, 3´ terminus VP1 gene sequence analysis (*8*) showed that they were E18 (n = 7), CBV3 (n = 1), and E16 (n = 2); 2 could not be typed with serologic or molecular methods because the 3´ terminus of VP1 gene amplification was negative. The analysis of the 3´ terminus of VP1 gene of the remaining 5 cerebrospinal fluid (CSF) and 3 nasopharyngeal isolates showed that they were similar to the recently proposed EV75 serotype (*5*). These 8 isolates were obtained from samples from children in Bilbao (n = 3), Granada (n = 3), Barcelona (n = 1), and the Canary Islands (n = 1). In 4 patients with aseptic meningitis, EV75 was isolated from CSF. EV75 was isolated from CSF of a fifth patient who had symptoms of fever and irritability. The remaining 3 EV75 isolates were from nasopharyngeal swabs of children who had fever, respiratory disease, or gastroenteritis. All isolates were grown in cell lines (rhabdomyosarcoma, lung adenocarcinoma, and human fetal lung fibroblast) and identified as EV by immunofluorescence with pan-EV antibody assays (Pan Entero Blend Chemicon, Temecula, CA, USA, and Monoclonal Mouse Anti-Enterovirus, Dako, Glostrup, Denmark).

Phylogenetic analysis of the isolates from 2005 was performed on the basis of complete VP1 gene sequence (GenBank accession nos. DQ468137–DQ468142). The 5´ terminal domain was obtained by reverse transcriptase–PCR with specific primers EV75_sense: 5´-GAAAGCTTYTTCCAAGGGGA-3´ and EV75_anti: 5´-GAGAAGTGKGACCAWCCATC-3´. Phylogenetic analysis of the Spanish isolates and representatives of all other species B EVs showed that the Spanish isolates clustered (bootstrap value 100, Figure) with strains USA/OK85-10362, ETH74-1341, USA/VA86-10363, USA/CT87-10364-5, OMA98-10366, and BAN00-10367-8 (accession nos. AY556063–AY556070), corresponding to the proposed EV75. The Spanish isolates constitute a subgroup (bootstrap value 100, Figure). The similarity between the Spanish cluster and other EV75 isolates was 82.8%–85.4% at the nucleic acid level. Although the entire VP1 sequence was not available for the isolates from 2006, the VP1 3´ terminal analysis showed the strains belonged to the same cluster.

**Figure:**
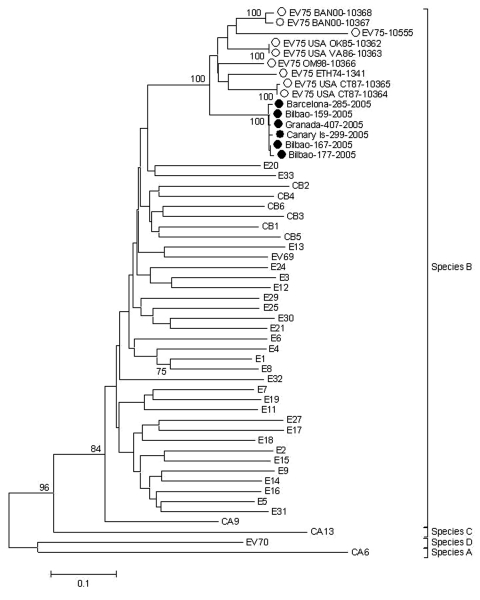
Phylogenetic analysis of complete VP1 sequences of Spanish enterovirus (EV) isolates (GenBank accession nos. DQ468137–DQ468142), the new proposed EV75 sequences (AY556063–AY556070 and AY919545), and prototype EV sequences (echovirus [E] 5, AJ241425; E31, AJ241435; E2, AF081315; E15, AJ241429; E14, AJ241428; E17, AF081330; coxsackie B virus [CBV] 2, AF081312; E26, AJ241433; E27, AF081338; E1, AJ241422; E8, AF081325; E4, AF081319; E21, AF081334; E30, AF081340; E25, AF081336; E29, AJ241434; CBV5, AF114383; CBV6, AF081313; E13, AF081327; EV69, AF081349; E24, AJ241432; E33, AF081346; E3, AF081316; E12, X77708; CBV3, M16572; CBV1, M16560; E6, AF081322; coxsackie A virus [CAV] 9, D00627; E16, AY302542; E9, AF524866; E7, AJ241426; E32, AF081345; E19, AJ241430; E11, AF081326; CBV4, X05690; E18, AF081331; E20, AJ241431; EV70, D17602; CAV6, AF081297; CAV13, AF081303; EV74, AY208118). Phylogenetic trees were constructed with the neighbor-joining method (MEGA version 3.0, available from http://www.megasoftware.net) with Kimura 2-parameter substitution model. Significance of phylogenies was estimated by bootstrap analysis with 1,000 pseudoreplicate datasets. Closed and open circles show Spanish and previously reported EV75 isolates, respectively.

To our knowledge, this is the first isolation of EV75 in Spain. Indeed, isolation of EV75 has not been reported in Europe. Given that the European EV75 isolate grows easily in a variety of cell lines, is detected by common EV genus-specific antibodies, and that EV surveillance and typing were performed in Spain since 1988 (*2*), EV75 might have begun to circulate in Spain recently. However, because isolates are not obtained from all aseptic meningitis patients and many EVs are detected by PCR but never typed, we cannot rule out the possibility of previous asymptomatic circulation.

The European strains of EV75 appear to represent a different evolutionary lineage than those previously described in the United States, Asia, and Africa (*9*). Only 1 of those EV75s was obtained from CSF (a nonspecific neurologic syndrome). Thus, EV75 has not been associated with aseptic meningitis, despite the fact that EV infections are a common cause of aseptic meningitis. Most of the Spanish isolates (5 of 8) were associated with aseptic meningitis in children. Although the number of EV75-associated cases was not high (as a percentage of the number of EVs isolated from aseptic meningitis patients, 10.8%), the wide distribution of the cases may indicate wide circulation. To avoid outbreaks of aseptic meningitis caused by previously noncirculating EVs (EV13, 2001 [10]) and to help define the extent of circulation of newly identified EV types, careful surveillance of aseptic meningitis should be undertaken.
